# The efficacy and safety of two different doses of caffeine in respiratory function of preterm infants

**DOI:** 10.22088/cjim.9.1.46

**Published:** 2018

**Authors:** Fatemeh Faramarzi, Mohammadreza Shiran, Mohammadreza Rafati, Roya Farhadi, Ebrahim Salehifar, Maryam Nakhshab

**Affiliations:** 1 Department of Clinical Pharmacy, Faculty of Pharmacy, Mazandaran University of Medical Sciences, Sari, Iran; 2 Immunogenetics Research Center, Faculty of Medicine, Mazandaran University of Medical Sciences, Sari, Iran; 3 Department of Pediatrics, Faculty of Medicine, Mazandaran University of Medical Sciences, Sari, Iran

**Keywords:** Caffeine, Extubation failure, CPAP failure, Preterm infants

## Abstract

**Background:**

Caffeine is widely used for prevention of apnea and helps successful extubation from mechanical ventilation. It facilitates the transition from invasive to noninvasive support and reduces duration of continuous positive airway pressure (CPAP) in preterm infants. The optimum caffeine dose in preterm infants has not been well-studied in terms of benefits and risks. We compared efficacy and safety of once versus twice-daily caffeine dose in premature infants.

**Methods:**

This study was a randomized clinical trial conducted in Bu-Ali Sina Teaching Hospital, Sari. Patients with gestational age of <37 weeks were included. Both groups received 20 mg/kg loading dose of caffeine intravenously followed by maintenance dose of 5 mg/kg/day in group 1 or 2.5 mg/kg every 12 hours in group 2. Extubation failure, CPAP failure and possibly adverse reactions were evaluated.

**Results:**

The mean of gestational age and birth weight were 32.27±3.23 (weeks) and 1824.5±702.54 (gr), respectively. The rate of extubation and CPAP failure and length of NICU stay were lower in twice-daily-group with no statistically significant difference. The means of O^2^ saturations on the first three days of caffeine therapy were higher in twice-daily-group. Caffeine was generally safe and well tolerated.

**Conclusions:**

This study, which assayed short-term effects of caffeine, showed that twice daily caffeine maintenance dose was related to more benefits in facilitating extubation or prevention of CPAP failure in preterm infants. However, there was not statistically significant difference between two groups.

Respiratory distress syndrome (RDS) is generally seen in preterm infants. RDS in neonates is managed with the intention to supply interventions that enhance survival as well as lessen potential complications, such as the risk of broncopulmonary dysplasia (BPD) (1-3). Many of the patients require mechanical ventilation and may be ventilator dependent for several days or even many weeks. The patients who have apnea and poor respiratory drive need further time of mechanical ventilation. Prolonged mechanical ventilation is associated with several short-term and long-term complications such as barotrauma and the development of chronic lung disease, atelectasis, air leak syndrome, pneumonia, neurodevelopmental impairments and bronchopulmonary dysplasia (4, 5). Methylxanthines (MGs) have been used 25-years ago as the backbone of pharmacologic treatments of respiratory disorders in premature infants. MGs are still widely used to manage apnea and facilitate successful extubation from mechanical ventilation (6-8). The efficiency of caffeine, as a preferred methylxanthine, to stimulate respiration has been well proven. 

Caffeine also, has significant favorable impact on neonatal morbidity such as BPD, patent ductus arteriosus ligation and so on. Useful effects, safety, efficacy, and cost-beneficial use caffeine introduced as the ‘silver bullet’ in neonatology (9). The results of previous studies revealed that caffeine enhances respiratory muscle strength and lung function followed by easier weaning of mechanical ventilation in premature infants (10). Also, a rapid and sustained increase in diaphragmatic activity and tidal volume was reported in preterm infants followed by caffeine administration (11). Previous studies have shown that caffeine citrate was generally well tolerated by premature neonates in clinical trials and declined the incidence of apnea in this population compared with placebo. 

It has established treatment and found to be equally effective like theophylline but has an overall superior safety due to a wider therapeutic index (12). Additionally caffeine is related to superior outcomes due to its lower toxicity and it is a preferred drug for apnea in preterm infants with respiratory problems (13). 

In a large randomized clinical trial, it has proven that the use of caffeine in very low birth weight infants reduced the incidence of BPD and duration of continuous positive airway pressure (CPAP) with no short-term adverse effects (14). The infants were followed-up until the age of five after caffeine therapy and established its long-term safety (15). Another report in 2006 indicated that infants in the caffeine group had a shorter duration of CPAP and mechanical ventilation than those in the placebo group and have lower incidence of BPD (16). 

The assessment of long-term effects of caffeine therapy in neonates showed an improvement in survival rate with no disability in neurodevelopmental status at 18 to 21 months in premature infants (17). Several reviews about caffeine in the treatment of premature infant respiratory disorders confirmed the overall advantages of this drug and explained it as the drug of choice in this condition with some protective effects on the brain and lungs with few side effects (18-23). Additionally, based on the recent multicenter and observational study to assess the clinical use, outcome and safety profile of caffeine in the treatment of apnea of prematurity (AOP), this drug was safe to use and the incidence of adverse drug reactions was low (24). In brief, caffeine has a significant function as a noninvasive respiratory support. It facilitates the transition from invasive to noninvasive support, reduces the duration of positive airway pressure support and decreases the risk of BPD in preterm infants. Nevertheless, the optimum caffeine dose in preterm infants with respiratory distress syndrome has not been well studied as well as heterogeneous reports on the optimal loading and maintenance dose of caffeine in several studies in terms of benefits and risks. Many investigations have been conducted about various dosing regimens in the improvement or prevention of respiratory disorders of premature infants (5, 25-31). These dosage regimens, although, have been associated with varying degrees of success. 

The current standard dosing regimen for caffeine citrate is 20 mg/kg (or 10 mg/kg as caffeine base) as a loading dose followed by 5mg/kg/day (or 2.5 mg/kg as caffeine base) as maintenance dose (32, 33). We hypothesized the 12-hour-interval leading to the more stable plasma drug concentrations and improving patient's outcome compared to once-daily dosing. The aim of this study was to compare efficacy and safety of once versus twice-daily caffeine-dose in premature infants with respiratory distress syndrome. 

## Methods

This study was an open-label randomized clinical trial conducted at a neonatal intensive care unit in Bu-Ali Sina Teaching Hospital, Sari, Iran (between July 2015 and August 2016). Study protocol was approved by the Research Ethics Committee of Mazandaran University of Medical Sciences, Sari, Iran. This trial was registered at IRCT.ir (reference number IRCT201510172342N4). All subjects' parents were informed about the nature and purpose of the study. This included an explanation of aims, methods, objectives, and potential hazards of the study and informed signed consent was obtained from their parents. The investigator explained to the parents that they were under no obligation to take part in the study and could withdraw at any time. Patients were included in the study if they had a gestational age of 26 to 37 weeks, with evidence of respiratory distress syndrome that was diagnosed by a neonatologist and treated by caffeine therapy facilitating earlier weaning process from mechanical ventilation or undergoing CPAP therapy. 

The exclusion criteria were asphyxia, hypoglycemia, intracranial ventricular hemorrhage (IVH), major congenital anomaly and previous exposure to methylxanthine therapy. If the patient met the inclusion criteria, he/she was enrolled and randomly assigned to one of the two study groups. Both groups received a 20 mg/kg loading dose (LD) of caffeine citrate that was administered intravenously over 30 minutes followed by a maintenance dose (MD) of 5 mg/kg every 24 in group 1 or 2.5 mg/kg infused every 12-hour-interval in group 2 over 20 minutes. Demographic characteristics (gestational age, gender, birth weight, type of delivery and Apgar score at 1 and 5 minutes after birth, age at initiation of caffeine, duration of caffeine therapy, length of stay in NICU, ventilator modality, primary RDS score, daily blood gas levels and routine laboratory tests) were recorded. Extubation failure, CPAP failure, duration of the ventilator and CPAP therapy for all patients were also registered as primary outcomes. In addition, an average of heart pulse rate and blood pressure based on the values registered in the daily sheets was recorded for all infants. 

The possible adverse drug reaction of caffeine including tachycardia, feeding intolerance, hyperglycemia or hypertension were also investigated in case they happen. Side effects and clinical worsening were used to assess safety and tolerability. The mentioned parameters were assessed since the time of caffeine therapy initiation until the infants treatment. All statistical analyses were conducted using SSPS Version 19 (SPSS Inc., Chicago, IL, USA) and a p-value ≤0.05% was considered statistically significant. Number and percent age are represented the qualitative variables and mean±SD displayed the quantitative variables. Continuous variables of two groups were compared using independent samples t-test, while qualitative variables were analyzed using chi-square test. 

## Results

**Figure 1:**
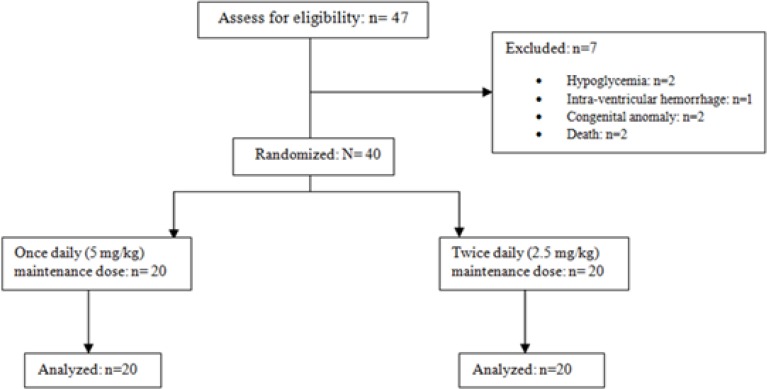
Diagram of the participants

**1. Demographic and drug dosing data analysis: **Forty-seven patients were enrolled in the study protocol and forty of them fulfilled the study criteria. Seven patients were excluded, of these, two patients were due to hypoglycemia, one with IVH, two had congenital anomaly and another two because of death (figure 1). Half of the patients (50%) were girls, the mean of gestational age was 32.3±3.2 weeks weeks, the mean of birth weight was 1824.5±702.54 gr and the type of delivery in majority (92.5%) of patients was cesarean (c/s). 

Demographic and clinical characteristics of the neonates in two groups are presented in table 1. There were no significant differences in gender, gestational age, birth weight, type of delivery, Apgar scores at 1 and 5 minutes after birth, RDS score, surfactant therapy (INSURE) and age at the beginning of treatment of the two groups. The average length of stay in NICU was 17.8±18.5 days and duration of caffeine therapy on average was 5.33±2.95 days, however, we considered the first three days of caffeine therapy in our analyses for all the patients. 

**Table1:** Demographic and clinical characteristics of the patients in two study groups

**Subject**	**All subjects** **(n=40)**	**Group 1** **(n=20)**	**Group 2** **(n=20)**	**P-value**
Female/male	20.20	11.9	9.11	0.52
GA at birth (week)	32.27±3.23	32.2±3.06	32.34±3.46	0.89
BW(gr)	1824.5±702.54	1772±675.27	1877±742.5	0.64
Vaginal /Cesarean delivery	3.3	1.19	2.18	0.54
Apgar, 1 min/ Median(IQR)	9 (8-9)	9 (8-9)	9 (9-9)	0.32
Apgar, 5 min/ Median(IQR)	10 (9-10)	10 (9-10)	10 (9.25-10)	0.86
RDS score/ Median(IQR)	6 (4-7)	6 (4.25-7)	5.5 (4-7)	0.49
Surfactant (INSURE)	19 (47.5)	12 (60)	7 (35)	0.11
PNA at initiation of caffeine (day)	8.23±9.2	7.1±7.7	6.7±7.2	0.86

GA:gestational age, BW: birth weight, Apgar1: Apgar score in 1th minutes after birth, Apgar5: Apgar score in 5^th^ minutes after birth, RDS: Respiratory distress syndrome score at the time of admission, PNA: postnatal age. Group1: once daily, group2: two divided dosing per day of caffeine.

**2. Effect of caffeine dose on the respiratory status of patients: **Twice a day compared to once daily dosing regimen of caffeine resulted in a reduction in failure of extubation and CPAP failure rate and length of NICU stay among preterm infants (14.7 VS 12 days, respectively), although they were not statistically significant. Nonetheless, there were no significant differences in the duration of mechanical ventilation, CPAP therapy and mortality rate between the two groups (table 2.(

**Table 2:** Comparison of neonatal outcomes among preterm infants receiving caffeine either in-group 1 (n=20) (once daily) or group 2 (n=20) (twice daily)

Parameters*	**Group 1**	**Group 2**	**Pvalue**
Extubation failure**	8(40%)	6(30%)	0.50
N-CPAP failure***	5(25%)	4(20%)	0.70
Rate of mortality	4(20%)	4(20%)	1
Duration of mechanical ventilation (days)	12.82±15.12	13.63±11.67	0.90
Duration of CPAP (days)	5.69±4.97	6.36±10.37	0.82
Length of NICU stay (day)	14.7±10.03	12±7.23	0.33

* value are presented in (n(%) or mean±SD)

** Extubation failure: (i) an inability to extubate from mechanical ventilation within 48 h of caffeine loading (ii) the use of reintubation within 7 days of commencing caffeine therapy

*** CPAP failure: SpO_2_<85%, PO_2_<50 mmHg, pH<7.2, PCO_2_>60 mmHg.

**3. Blood gas levels change during the course of caffeine therapy: **Arterial blood gas (ABG) parameters were measured daily for each patient and their mean values are presented in table 3. All pH values were in the normal range except the first day in group 1 that elevated to the normal range on the second day of treatment (from 7.33 to 7.37). In addition, all values of Pco_2_ and HCO3 in patients of group 2 were in normal range compared to group1 during the first 3 days of caffeine therapy. At any rate, these differences were not statistically significant. Most of the differences were seen in the values of the O^2^ saturation between two groups.

**Table 3:** Comparison of blood gas values between the two groups in the first 3 days of treatment with caffeine. All values are displayed on (Mean±SD)

**Parameters**		**Group 1**	**Group 2**	**Pvalue**
pH	Day 1	7.33±0.1	7.37±0.08	0.21
	Day 2	7.37±0.12	7.37±0.06	0.85
	Day 3	7.37±0.16	7.39±0.12	0.68
PCO2 (mmHg)	Day 1	51.9±21.2	43.3±14.3	0.14
	Day 2	46.8±19.2	42.8±11.1	0.42
	Day 3	50.2±31.6	45.5±18.7	0.57
HCO3 (mEq/l)	Day 1	28.4±8.1	25.3±6.5	0.19
	Day 2	28.9±8.3	26.1±4.9	0.18
	Day 3	27.1±5.8	26.2±5.9	0.63
O2 saturation (%)	Day 1	89.9±15.1	93.8±15.5	0.42
	Day 2	89.1±11.2	95.2±7.4	0.049*
	Day 3	89.1±14.6	95.9±5.5	0.056

* There was a significant difference in O2 saturation on day 2 between two groups.

All of O^2^ saturation levels in neonates that received caffeine twice a day were higher compared to those with once-daily-dosing even the difference on the 2^nd^ day was significant (p<0.05). Furthermore, the value of O^2^ saturation on the 3nd day moved towards a meaningful trend (P<0.05).

**4. Safety and Tolerability: **Heart rate, blood pressure, feeding intolerance and blood sugar of all infants were recorded daily based on the infants' laboratory tests or data in the NICU sheets. The mean values over the course of caffeine therapy have been shown in table 4. Hyperglycemia and hypertension episodes were lower in preterm infants that received caffeine twice a day compared to those with once-daily-dose. Hypertension was the most frequently reported adverse effect (AE) that occurred in some cases over the treatment. The mean arterial pressure had the greatest difference between two groups of neonates with the trend toward statistical significance (p=0.07).

Totally, intravenous administration of caffeine was generally safe and well tolerated. None of the patients had tachycardia during the study. Most of the AEs were reported as mild in severity, and no serious AEs were recorded. Eight deaths occurred during the study (4 cases in each group) of which they were not caffeine-related.

**Table 4:** Frequency and type of adverse effects observed following administration of caffeine

**Pvalue**	**Group2**	**Group1**	**Type of AE**	
0.56	1	2	Feed intolerance	
0.10	21(38.9%)	33(61.1%)	Hyperglycemia*	
0.22	35(43.2%)	46(56.8%)	Systolic BP	Hypertension**
0.63	34(42.2%)	38(52.8%)	Diastolic BP	
0.07	37 (39.4)	57(60.6%)	MAP	

* Blood glucose >125 mg/dl,

** Systolic blood pressure >75 mmHg, Diastolic blood pressure >45 mmHg, MAP>55 mmHg

## Discussion

Caffeine is one of the most currently used medications in the neonatal care units. Despite its widespread use in preterm infants, there has been little information about optimal dose in these patients. This clinical trial was designed to assess the efficacy, safety and short-term effects of two different dosing regimen of caffeine citrate in periextubation management of preterm infants with gestational age <37 weeks. In sum, 40 preterm infants were included in this study and received either caffeine 2.5 mg/kg twice daily or 5 mg/kg once daily. All of them received caffeine 20 mg/kg as a loading dose in the beginning of treatment. The results indicated that caffeine improved the respiratory function of neonates in two study groups. There was a trend to a benefit for patients receiving caffeine 2.5 mg/kg twice daily compared to the 5 mg/kg once daily dosing; yet there was not statistically significant difference. The rate of extubation and CPAP failure and length of NICU stay were lower in the twice-daily-group than once-daily-group, too. In addition, the means of O^2^ saturations on the first three days of treatment with caffeine were higher in group 2 compared to group 1. Thus the mean difference of O^2^ saturation between two groups was meaningful on the second day (p<0.05) and a trend towards significant on the third day (p<0.05). 

Previous studies indicated the efficacy of caffeine in the improvement of respiratory function in preterm infants with low birth weight. Additionally, there are many reports of caffeine useful effects on the treatment or prevention of apnea in premature infants. Caffeine is an effective respiratory stimulant drug in the premature infants (26). This drug significantly decreases duration of mechanical ventilation and need for it (34). Caffeine led to a significant decrease of apnea in the treatment group as compared with the control group with no adverse effects during the study (35). Besides, this drug improves tidal volume ventilation and mean inspiratory flow mostly by stimulating central respiratory drive (36). 

A study has reported that the use of caffeine can lead to prevent apnea episodes that need intervention and also enhances the results of pneumogram (37). A randomized double-blind clinical trial was conducted by Steer et al. that compared three dosing regimens of caffeine (3, 15 or 30 mg/kg) for periextubation control of premature infants. This trial revealed that the infants in higher dose group had lower apnea events through the week after extubation. Likewise, the short term safety of caffeine was endorsed (30). Other trials compared two different doses of caffeine (5 or 20 mg/kg/day) for extubation of preterm infants that was showed short-term effects of higher-dose regimen in the periextubation days. A dose of 20 mg/kg/day was administered in this period eases extubation, declines the time of mechanical ventilation and decreases apnea events after extubation without the increase of short-term side effects (5). The results of a multicentre, randomized and controlled trial that compared two different dosing regimens of caffeine in preterm infants showed that the high-dose of caffeine as (20 mg/kg/day) reduces the need for respiratory support versus the standard dose (5 mg/kg/day) with no adverse outcomes in two years of age (38). 

Extended caffeine therapy causes the lower rate and severity of intermittent hypoxia in premature infants (39). The preventive effect of caffeine on apnea has been proven when it is administered in high-risk premature infants (40). The prophylactic use of caffeine results in the lower rate of mortality or BPD and PDA with no adverse outcomes (41). It has also been shown the favorable effect of caffeine on the treatment of central apnea is via stimulated neural breathing (42). The results of a randomized controlled trial revealed that the administration of high dose caffeine (loading 40 mg/kg then maintenance dose of 20 mg/kg/day) compared with low dose caffeine (loading 20 mg/kg then maintenance dose of 10 mg/kg/day) decreased the rate of extubation failure in ventilated infants and apnea episodes without serious adverse effects (28). Findings of a recent study that have compared two different doses of caffeine in treatment of primary AOP demonstrated that the patients in high caffeine doses (LD: 20 mg/kg and MD: 15 mg/kg/day) had more benefits than the low caffeine doses (LD: 20 mg/kg and MD: 5 mg/kg/day) with no further side effects (31). Besides, caffeine notably improved the early pulmonary function and decreased apnea (43). 

In the current study, lower frequency of short-term adverse effects of caffeine was observed in twice-daily-dose group compared to single-daily-dose group. This issue may be due to the stable concentrations and lower plasma peak levels of caffeine following the twice-daily-dose administration. Closely similar to our results, previous studies which compared different dosing regimens of caffeine determined that low dose caffeine was related to fewer side effects. For example after 10 mg/kg loading dose of caffeine, the maintenance dose of 5 or 2.5 mg/kg was administered to groups 1 and 2, respectively. The results indicated that caffeine significantly reduces the apnea episodes in both groups and the rate of adverse effects such as tachycardia and feeding intolerance in group 2 was notably lower than in group 1 (29). A 20 mg/kg followed by 5 mg/kg/day caffeine citrate administered intravenously for 10 days has been shown to be safe and effective for treating apnea in premature infants of gestational age 28–32 weeks (44). The results of this research showed a trend to a partial superiority efficacy for infants receiving a dose of 2.5 mg/kg twice daily compared to 5 mg/kg once daily. Despite more improvements in the clinical outcome of patients in group 2, no significant difference was observed between the two groups. It may result in meaningful outcomes if the drug use in a clinical trial has larger sample size. These beneficial effects in twice-daily maintenance dose group may be due to a more steady state plasma level of caffeine in infants. 

This study which assayed the short-term effects of caffeine therapy, concludes that twice versus once-daily caffeine maintenance dose was related to more benefits in facilitating extubation or prevention of CPAP failure in infants of born gestational age less than 37 weeks with no statistically significant difference between two groups.
